# Diagnostic value of transthoracic needle biopsy in lung tumors

**DOI:** 10.1590/1806-9282.20231082

**Published:** 2024-04-22

**Authors:** Ozlem Sogukpinar, Ulku Aka Akturk, Makbule Ozlem Akbay, Erdal Tatlidil, Dilek Ernam

**Affiliations:** 1 University of Health Sciences, Sureyyapasa Chest Diseases and Thoracic Surgery Training and Research Hospital, Department of Chest Diseases – İstanbul, Turkey.; 2 Denizli State Hospital, Department of Chest Diseases – Denizli, Turkey.

**Keywords:** Biopsy, fine-needle aspiration, Thoracic neoplasms, Interventional ultrasonography, Cytopathology

## Abstract

**OBJECTIVE:**

Thoracic ultrasonography is widely used in imaging peripheral lesions and invasive interventional procedures. The aim of this study was to assess the diagnostic value of thoracic ultrasonography-guided transthoracic needle aspiration biopsy and the factors affecting the diagnosis of peripheral tumoral lung lesions.

**METHODS:**

The lesion size, biopsy needle type, number of blocks, complications, and pathology results were compared in 83 patients between January 2015 and July 2018. The cases with pathological non-diagnosis and definite pathological diagnosis were determined. For the assessment of the factors affecting diagnosis, the size of the lesions and the biopsy needle type were evaluated. Biopsy preparations containing non-diagnostic atypical cells were referred to a cytopathologist. The effect of the cytopathological examination on the diagnosis was also evaluated.

**RESULTS:**

Pathological diagnosis was made in 66.3% of the cases; cell type could not be determined in 22.9% of the cases, and they were referred to a cytopathologist. After the cytopathologist's examination, the diagnosis rate increased to 80.7%. Diagnosis rates were higher when using tru-cut than Chiba and higher in cases with tumor size >2 cm than smaller.

**CONCLUSION:**

Thoracic ultrasonography-guided transthoracic needle aspiration biopsy is a preferred approach to the diagnosis of peripheral tumoral lung lesions, given its high diagnostic rate, in addition to being cheap, highly suitable for bedside use, and safe, and the lack of radiation exposure.

## INTRODUCTION

The most non-invasive and safest method should be preferred for the tissue diagnosis of lung lesions, considering the lesion localization, the patient's pulmonary function capacity, and the availability of diagnostic resources. Based on the method of sample collection, biopsies can be planned as bronchoscopic, percutaneous, thoracoscopic, or surgical. An imaging modality guides percutaneous biopsies.

Transthoracic needle aspiration biopsy (TTNAB) procedures have improved over time in line with advances in imaging technologies^1^, the improvement of punction needles, and the improvement of cytological examination methods^2^. TTNAB can be performed under the guidance of ultrasonography (USG) or computerized tomography (CT)^3,4^, fluoroscopy, or magnetic resonance imaging (MRI), with CT-guided TTNAB being the most commonly used approach^3,5^. USG-guided TTNAB is preferred for lesions attached to the chest wall that are of sufficient size^6^.

The advantages of thoracic USG over other imaging methods include its low expense, portability, repeatability, bedside applicability, and absence of radiation exposure during the procedure^7,8^. Thoracic USG guidance is used by pulmonologists during various pulmonary procedures, including thoracentesis, chest tube placement, transthoracic aspiration, and biopsies^9^, in that it allows the visualization of needle placement and movement during biopsies^5^, while other advantages include the opportunities presented for real-time biopsy, the multi-dimensional images, which allow the lesion to be approached from different angles, and the ability to make a dynamic evaluation of proximity to vascular structures^10^.

Thoracic USG eases access to peripheral lesions attached to the chest wall, and USG-guided TTNAB provides similar diagnostic accuracy and safety to CT-guided TTNAB, in addition to reducing the time needed for the biopsy^11^. Complication rates are also lower than with CT-guided TTNAB^12^. USG-guided TTNAB is a minimally invasive procedure that is safe^12,13^ and fast^14^ and offers high diagnostic accuracy^8,15–17^.

Based on these advantages, USG-guided biopsy should be the first choice of clinicians for eligible patients to diagnose peripheral lung lesions.

In this study, we investigate the diagnostic value of thoracic USG-guided TTNAB in patients with peripheral tumoral lesions and the factors affecting it.

## METHODS

We carried out a retrospective review of the patients who underwent thoracic USG-guided TTNAB in the pulmonology clinics between January 2015 and July 2018. Excluded from the study were patients with missing information. In the review of the medical files of the patients who underwent biopsy, information including demographics, the size of the peripheral lesion, the biopsy needle type, the number of blocks, and the pathological diagnosis were all recorded. All biopsies were guided by the same USG device (Mindray, North America) using a 3.5–5 MHz convex probe, a 22-gauge Chiba needle, and a tru-cut biopsy needle. For the assessment of the factors affecting diagnosis, the lesions were classified into two groups: those larger and those smaller than 2 cm, considering the lesion size and the biopsy needle type.

Cases in which no diagnosis could be made after a pathological examination were considered non-diagnostic, while those with a definitive pathological diagnosis were considered diagnostic. Preparations including atypical cells but without type confirmation were referred to a cytopathologist, and the impact of the cytopathological examination on the diagnosis was recorded. We also recorded any complications reported in the medical records of the patients who were followed up after undergoing biopsy. Pneumothorax was evaluated with chest radiographs taken immediately in symptomatic patients and 2 h after the procedure in asymptomatic patients.

The study was designed by the International Helsinki Declaration, and institutional ethics committee approval was granted for the continuation of the present study (116.2017.R-250).

### Statistical analysis

The Statistical Package for the Social Sciences 22.0 version (IBM Corporation, Armonk, New York, USA) package program was used for the analysis of the data. Descriptive statistics were used to present baseline characteristics. Continuous variables were expressed as mean±standard deviation and median (range). Qualitative data were calculated as percentages. Chi-square test (^2^) was used to compare the differences between groups. Student's t-test was used in case of normal distribution of variables. Mann–Whitney U test was used to compare parameters that did not show normal distribution. A p<0.05 was considered statistically significant.

## RESULTS

The files of 110 patients who underwent thoracic USG-guided biopsy on the specified dates were examined, and 83 patients with complete data were identified. The mean age of the patients was 61.2±9.8 years, and 62.7% (n=52) were male.

While a definitive pathological diagnosis was made in 66.3% (n=55) of the patients, the type could not be confirmed in 22.9% (n=19) despite the detection of atypical cells. Preparations containing atypical cells but not a diagnostic type were referred to a cytopathologist and examined. Among the 19 patients evaluated by a cytopathologist, 12 (68%) were given a definitive diagnosis, while the subtype could not be identified in 7 patients (32%). The diagnosis rate increased from 66.3 to 80.7% in patients with atypical cells referred to a cytopathologist. The diagnostic rate of USG-guided biopsy was thus found to be 80.7%.

The rate of diagnosis based on biopsies performed using a tru-cut needle was higher than that of those performed using a Chiba needle (p=0.04). In contrast, the rate of diagnosis did not differ depending on the number of blocks. The diagnosis rate was higher for tumors larger than 2 cm (p=0.03). Table 1 presents the distribution of the pathological findings after a diagnostic examination, while a comparative analysis of the diagnosis rates associated with biopsy needle, tumor size, and number of blocks is presented in Table 2.

**Table 1:** Distribution of the pathological findings after a diagnostic examination.

Distribution of diagnoses (n=83)	
Adenocarcinoma	27 (32.6%)
Squamous cell carcinoma	27 (32.6%)
Non-small cell carcinoma	14 (16.8%)
Small-cell carcinoma	7 (8.4%)
Adenocarcinoma with lepidic growth	3 (3.6%)
Neuroendocrine tumor	3 (3.6%)
Organized pneumonia	1 (1.2%)
Sarcoidosis	1 (1.2%)

**Table 2:** A comparative analysis of the diagnosis rates associated with biopsy needles, tumor sizes, and number of blocks.

		Rate of diagnosis (%)
**Biopsy needle**		
	Tru-cut	88.4
	Chiba	78.2
**Tumor size**		
	<2 cm	65.3
	≥2 cm	86.6
**Number of blocks**		
	1	77.6
	≥2	84.3

None of the pathological examinations led to a diagnostic outcome in 10.8% (n=9) of the patients, for whom more invasive procedures were needed.

Among the major complications, one patient developed pneumothorax and required chest tube placement, and another experienced bleeding in the lesion with consolidation around the mass.

## DISCUSSION

It is found in the present study that thoracic USG-guided TTNAB is associated with high diagnostic and low complication rates in patients with peripheral lung lesions. The rate of diagnosis using thoracic USG-guided TTNAB was higher for tumors with diameters larger than 2 cm than for smaller tumors. Moreover, biopsies performed using tru-cut needles were associated with a higher diagnosis rate than those conducted with a Chiba needle. A cytopathologist was consulted for the cases with atypical cells identified in the pathological examination but in which no subtype classification could be made, which led to a definitive diagnosis in some of the cases, increasing the overall diagnosis rate.

The diagnosis rate with USG-guided TTNAB varies between 71.8 and 88.7% in the literature, depending on the malignancy potential of the lesion, the originating tissue, the size of the lesion, the presence of necrosis, and the biopsy needle used^8,15–17^. Consistent with the literature, the rate of diagnosis with thoracic USG was found to be 80.7% in this study.

Another important issue is the selection of the biopsy needle and the gauge. Conflicting results have been reported regarding the diagnostic effect of larger-diameter needles^18,19^. Diacon et al., reported diagnostic accuracy rates of 82 and 76% for USG-guided TTNAB and cutting-needle biopsy, respectively, and that the combination of them increases diagnostic accuracy to 89%^13^. In another study, Dogan et al., reported diagnostic accuracy rates of 71.8% with USG-guided TTNAB and 81.2% with tru-cut biopsy, and that the diagnostic accuracy rate increased to 93.7% when combined^15^. In this study, the diagnostic rate with TTNAB using a Chiba needle was 78.2 and 88.4% when the tru-cut-needle biopsy method was used, representing a significant difference (p=0.04). There are also studies in which no difference was found between cutting needles and TTNAB. A systematic review of 11 studies found no significant difference between biopsy needles^20^, and there is still no standard approach to needle selection in thoracic USG-guided TTNAB.

Small lesions can be challenging in thoracic USG-guided TTNAB as they are dynamic during respiration, and the biopsy needle will have a smaller range of motion. Guo et al., reported lower diagnostic rates in lesions smaller than 2 cm when compared to larger lesions and lower rates in lesions larger than 5 cm due to necrosis^18^. Huang et al., reported thoracic USG-guided biopsies to be appropriate and safe for lesions smaller than 2 cm and that biopsies using cut-needle methods were 3.4 times more diagnostic than thin-needle biopsy approaches^21^. In our study, diagnostic accuracy was higher for lesions larger than 2 cm than for smaller lesions (p=0.03). As lesions get smaller, inserting the needle into the lesion becomes more challenging, and the amount of collected biopsy material is limited. While small lesion size is not an obstacle for thoracic USG-guided TTNAB, it is essential that the needle be placed inside the lesion and sufficient biopsy material be collected. Thoracic USG is associated with a higher diagnostic accuracy rate in larger lesions, as it allows multidimensional access, but it is also a safe and convenient biopsy guide with a diagnostic accuracy rate that cannot be underestimated in lesions smaller than 2 cm. As USG allows real-time biopsy, the respiration-associated movements of smaller lesions can be easily managed.

The number of transitions in biopsies is not defined; it is determined by factors like lesion accessibility, complication risk, sample quality, needs for real-time pathology examination, and sample sufficiency^3^. Lee et al., reported that fewer transitions would be needed during thoracic USG-guided biopsies than with CT-guided biopsies, which they attributed to the efficacy of needle placement and the collection of sufficient samples that real-time imaging USG allows^5^. In this study, we found that the number of blocks collected during biopsy made no significant difference for pathological diagnosis, with high rates of diagnosis reported with both single and double blocks of 77.6 and 84.3%, respectively.

The data pooled from 10 studies before 2015 investigating thoracic USG-guided TTNAB showed that the most common complication was pneumothorax, occurring in 4.4% of cases, while the same was much higher (20.5%) when BT-guided TTNAB was used^17^. Another study reported the overall complication rate to be lower when using USG-guided TTNAB (7%) when compared to CT-guided TTNAB (24%)^5^. A lower complication rate can be expected with thoracic USG-guided TTNAB than with CT-guided TTNAB since there is no lung tissue between the probe and the lesion when using USG, and therefore no lung tissue is invaded during the procedure. Visualizing the lesion would otherwise not be possible with USG. Huang et al., reported bleeding and pneumothorax complication rates of 6.7 and 2.1%, respectively^21^. In this study, only one patient developed pneumothorax requiring chest tube placement, while one patient developed bleeding into the lesion with consolidation around the mass, and both were successfully managed. The findings of the present study support the use of thoracic USG-guided TTNAB as a safe procedure for the diagnosis of peripheral lung lesions and its association with a low rate of complications. Another advantage of USG-guided biopsy is that it allows the detection of such complications as pneumothorax immediately after the procedure. The sliding sign in thoracic USG, meaning the sliding of visceral pleura and parietal pleura over each other, can successfully exclude pneumothorax formation^22^. In this study, sonographic controls were made to check for pneumothorax after each biopsy.

This study was limited by its single-center, retrospective design. Furthermore, the number of patients was limited, and the biopsy needle and method were selected in the light of the available radiological findings by the interventional pulmonologist performing the procedure, preventing randomization.

## CONCLUSION

Based on the findings of the present study, thoracic USG-guided TTNAB can be considered a safe, fast, and effective diagnostic method for peripheral lung lesions that can be visualized by thoracic USG. Due to the advantages described, experienced centers should make more frequent use of thoracic USG for the diagnosis of peripheral lung lesions.
